# LIFESTYLE INDICATORS AND CARDIORESPIRATORY FITNESS IN ADOLESCENTS

**DOI:** 10.1590/1984-0462/;2017;35;1;00016

**Published:** 2017

**Authors:** Eduardo Rossato de Victo, Gerson Luis de Moraes Ferrari, João Pedro da Silva, Timóteo Leandro Araújo, Victor Keihan Rodrigues Matsudo

**Affiliations:** a Centro de Estudos do Laboratório de Aptidão Física de São Caetano do Sul, São Caetano do Sul, SP, Brasil.

**Keywords:** Lifestyle, Physical fitness, Motor activity, Adolescent

## Abstract

**Objective:**

To evaluate the lifestyle indicators associated with cardiorespiratory fitness in adolescents from Ilhabela, São Paulo, Brazil.

**Methods:**

The sample consisted of 181 adolescents (53% male) from the Mixed Longitudinal Project on Growth, Development, and Physical Fitness of Ilhabela. Body composition (weight, height, and body mass index, or BMI), school transportation, time spent sitting, physical activity, sports, television time (TV), having a TV in the bedroom, sleep, health perception, diet, and economic status (ES) were analyzed. Cardiorespiratory fitness was estimated by the submaximal progressive protocol performed on a cycle ergometer. Linear regression models were used with the stepwise method.

**Results:**

The sample average age was 14.8 years, and the average cardiorespiratory fitness was 42.2 mL.kg^-1^.min^-1^ (42.9 for boys and 41.4 for girls; *p*=0.341). In the total sample, BMI (unstandardized regression coefficient [B]=-0.03), height (B=-0.01), ES (B=0.10), gender (B=0.12), and age (B=0.03) were significantly associated with cardiorespiratory fitness. In boys, BMI, height, not playing any sports, and age were significantly associated with cardiorespiratory fitness. In girls, BMI, ES, and having a TV in the bedroom were significantly associated with cardiorespiratory fitness.

**Conclusions:**

Lifestyle indicators influenced the cardiorespiratory fitness; BMI, ES, and age influenced both sexes. Not playing any sports, for boys, and having a TV in the bedroom, for girls, also influenced cardiorespiratory fitness. Public health measures to improve lifestyle indicators can help to increase cardiorespiratory fitness levels.

## RESUMO

**Objetivo:**

Associar indicadores de estilo de vida com a aptidão cardiorrespiratória em adolescentes de Ilhabela, São Paulo.

**Métodos:**

A amostra foi composta de 181 adolescentes (53% meninos) que faziam parte do Projeto Misto-Longitudinal de Crescimento, Desenvolvimento e Aptidão Física de Ilhabela. Foram analisados: composição corporal (peso corporal, estatura e índice de massa corpórea, ou IMC), transporte para escola, tempo sentado, atividade física, prática de esportes, tempo de televisão (TV), TV no quarto, sono, percepção de saúde, dieta e nível econômico (NE). A aptidão cardiorrespiratória foi estimada por meio do protocolo progressivo submáximo realizado em um ciclo ergômetro. Foram utilizados modelos de regressão linear com o método *stepwise*.

**Resultados:**

A média de idade da amostra foi de 14,8 anos e a de aptidão cardiorrespiratória foi de 42,2 mL.kg^-1^.min^-1^ (42,9 nos meninos e 41,4 nas meninas; *p*=0,341). Na amostra total, IMC (coeficiente de regressão não estandardizado - B=-0,03), estatura (B=-0,01), NE (B=0,10), sexo (B=0,12) e idade (B=0,03) foram significativamente associados com aptidão cardiorrespiratória. Nos meninos, o IMC, a estatura, não praticar nenhuma atividade esportiva e a idade mostraram associações significativas com a aptidão cardiorrespiratória. Já nas meninas, IMC, NE e TV no quarto associaram-se com aptidão cardiorrespiratória.

**Conclusões:**

Indicadores de estilo de vida influenciaram a aptidão cardiorrespiratória, sendo o IMC, o NE e a idade comum entre os sexos. Não praticar esportes, para os meninos, e ter TV no quarto, para as meninas, também influenciaram a aptidão cardiorrespiratória. Medidas de saúde pública para melhorar os indicadores do estilo de vida podem ajudar a aumentar os níveis de aptidão cardiorrespiratória.

## INTRODUCTION

Physical fitness is the ability to perform daily physical work without harming biological, psychological, or social health. One of the components of physical fitness is cardiorespiratory fitness (VO2max),^1^ measured in absolute values (L.min^-1^) and fitness relative to body mass (mL.kg^-^1.min^-1^). Low cardiorespiratory fitness values are positively associated with an overweight and sedentary lifestyle.^2^ In addition, there is a negative association of cardiorespiratory fitness with cardiovascular risk factors (for example: obesity, hypertension, hypertriglyceridemia),^3^ ^,^ ^4^. Cardiorespiratory fitness is also used to compare the physical fitness of students showing different nutritional statuses.^2^

Research has shown a decrease in the average values of cardiorespiratory fitness in students from several countries, including Brazil.^5^ ^,^ ^6^ In a 30-year analysis (1978/1980-2008/2010), Ferrari *et al* ^5^ demonstrated a significant reduction of cardiorespiratory fitness in normal weight students (defined as Z score of body mass index, or BMI, between -1 and 1) and overweight (Z score of BMI>1), and cardiorespiratory fitness being higher in normal weight students than in those who are overweight (mL.kg^-1^.min^-1^:-25.8% *versus* -16.2%). Another study conducted between 1998 and 2004 found that British children had increased BMI and decreased cardiorespiratory fitness.^6^

In addition to the association of cardiorespiratory fitness with body size, Aires *et al* ^7^ showed other relationships with many lifestyle indicators such as physical activity (PA), television time (TV), and adiposity. Moreover, cardiorespiratory fitness was also associated with anthropometric variables (waist-to-height ratio,^8^ obesity, and BMI).^9^ Sleep duration and dietary habits are also important aspects of lifestyle.^10^ Cuenca-Garcia *et al*,^11^ in a study conducted with adolescents from eight European countries, found that high levels of cardiorespiratory fitness are associated with higher intake of dairy products and breads/cereals in boys and lower consumption of sugary drinks in girls.

As cardiorespiratory fitness is related to metabolic syndrome^3^ ^,^ ^4^ and mortality,^12^ identifying lifestyle indicators as predictors in a single research study during the obesity epidemic may suggest the need for interventions and public health messages in order to change adolescent lifestyles. Therefore, this study intended to determine whether there is an association of lifestyle indicators with cardiorespiratory fitness in adolescents from Ilhabela, Sao Paulo.

## METHOD

This is a cross-sectional study, which is integrated in the Mixed Longitudinal Project for Growth, Development, and Physical Fitness of Ilhabela. The *Centro de Estudos do Laboratório de Aptidão Física de São Caetano do Sul* (CELAFISCS), São Paulo has been developing this project since 1978. Health professionals carry out all assessments. These professionals were previously trained on three consecutive days to collect data on physical fitness, PA, and eating habits of children aged 7 years and older, by means of tests and standard measurements. Methodological details, data collection, and additional information have been previously published.^5^ This project was approved by the Ethics Committee of the *Universidade Federal de São Paulo* (UNIFESP) under the Protocol number 0056/10.

Ilhabela is located on the north coast of São Paulo (Brazil). It has a land area of 348 km^2^ and population estimated at 32,782 inhabitants. Currently, Ilhabela has 4,430 students enrolled in primary education and 1,320 in high school.^13^

Adolescents participating in this study were selected by convenience criteria and by integrating the Mixed Longitudinal Project for Growth, Development, and Physical Fitness of Ilhabela. A database with 413 adolescents participating in the project between 2011 and 2014 was analyzed to compose the study sample. Among those adolescents, 181 met the following inclusion criteria:

- Aged 11 to 18 years.
- Having a completed full physical assessment (body weight, height, and aerobic power).
- Being regularly enrolled in the municipal school system.
- Having no clinical or functional limitations to perform the exercise test.
- Having informed consent forms signed by those responsible.

To assess cardiorespiratory fitness, aerobic power was estimated through the progressive submaximal test in a mechanical cycle ergometer (Monark^®^ Ergomedic 828E model). The test lasted eight minutes, with a warm-up load (four minutes) and a workload calculated based on body weight (four minutes). Blood pressure and heart rate values, and subjective perceived exertion were measured at rest and every minute of the test. Adolescents were asked to maintain a pedal rate of 50 revolutions per minute (RPM), and the bicycles were previously calibrated before the evaluations. Cardiorespiratory fitness (VO_2max_) was calculated in absolute (L.min^-1^) and relative (mL.kg^-^1.min^-1^) values. The Astrand nomogram was used, considering the heart rate of the last minute of the exercise load.^14^ The relative values were used for the analysis.

Body weight (kg) was measured by digital scale (Filizola^®^, Personal Life model), with the adolescent standing erect, with his or her back to the scale, with lateral distance of the feet and looking straight ahead. Height (cm) was determined using a stadiometer with a fixed base and mobile cursor, with the adolescent in the standing position, with his or her feet together. BMI (kg/m^2^) was calculated, and adolescents were classified as underweight: ≤-2 standard deviation (SD); normal weight: <-2 to 1 SD; overweight >1 to 2 SD; and obese: >2 SD, according to the reference data from the World Health Organization (WHO).^15^

Time spent sitting and PA were calculated using the short version of the International Physical Activity Questionnaire (IPAQ).^16^ Total sitting time (min/day) during one day of the week and the total sitting time on the weekend were requested. Adolescents were also evaluated according to the frequency and duration of moderate and vigorous PA and any walking done for at least ten minutes in the last week.^17^ For each of the domains assessed, scores of PA were determined by multiplying the weekly frequency by the duration of the activity when it was performed. Vigorous PA was multiplied by two and added to the scores of walking and moderate PA to obtain full PA score. Adolescents were classified into active (≥300 min/week of PA) or insufficiently active (<300 min/week of PA), as recommended by the WHO.^18^

The participants responded about transportation to school, activities performed, television (TV) time, TV in the bedroom, quality and quantity of sleep, perception of health, and diet, using the Diet and Lifestyle Questionnaire.^19^ For the type of transportation to school, the answers were: walking; bicycle, skates, skateboard, or scooter; bus, train, subway, boat; car or motorcycle; other. Responses were categorized into active and passive transportation. The adolescents also informed the commute time to school as follows: <5; 5-15; 16-30; 31-60; <60 minutes.^19^

For the amount of TV time, the following question was asked: On a school day, how many hours do you watch TV? Response options were none; ≤1; 2; 3; 4; ≥5 hours. The adolescents were categorized into <2 or ≥2 TV hours/day.^20^ Whether there was a TV present in the bedroom was also asked.^19^

Regarding the type of activities or sports practiced in the past 12 months, the responses were team sports; dance/martial arts classes; art/music classes; none of these alternatives.^19^ In regard to the sleep duration, adolescents reported the time they went to bed and woke up on weekdays and were classified into <8 or ≥8 hours/day.^21^ The quality and quantity of sleep were classified as very good, fairly good, fairly bad, and very bad.^19^ The perception of health was assessed according to the following: excellent, very good, good, fair, and poor.

The adolescents completed the questionnaire related to the consumption of 23 food items in a typical week.^19^ To identify existing food habits, principal component analysis (PCA) was used and considered the food items as input variables. PCA was conducted with orthogonal varimax transformation to maintain factors uncorrelated and to improve the interpretation. Two factors were identified: unhealthy dietary habits (fast-food*,* French fries, ice cream, sweets, pies, etc.) and healthy dietary habits (vegetables, oranges, fruit juices, fruit, etc.).^19^ The two scores were analyzed separately and treated as continuous variables. Higher values for each score represent unhealthy or healthy dietary habits, respectively.

Economic status (ES) was determined by a questionnaire developed by the *Associação Brazileira de Empresas de Pesquisa* (ABEP),^22^ and the questions were related to the quantities of color TVs, radios, bathrooms, automobiles, salaried employee, washing machines, VHS or DVDs, refrigerators, and freezers*.* The answers were 0; 1; 2; 3; 4; ≥5. With regard to the educational level of the head of the household, the answers were the following: did not study; 1st, 2nd, or 3rd grade; completed 4th, 5th, 6th, or 7th grade; completed the 8th grade; 1st or 2nd year of high school; completed the 3rd year of high school; bachelor’s degree. The calculation of the ES was done by a points system adopted as Economic Classification Standard Criteria in Brazil. The total score ranged from 0 to 46 and was classified as classes A=35-46; B=23-34; C=14-22, D=8-13, and E=≤7.

Statistical analysis was performed with the Statistical Package for Social Sciences (SPSS), version 22. For comparison between the sexes, Student’s t-test or chi-squared test were used. Linear regression models were applied (backward stepwise method) to identify lifestyle indicators associated with cardiorespiratory fitness. For the selection of variables, we considered *p*<0.05. The normality of the dependent variable was tested with the Kolmogorov-Smirnov test, and assumptions concerning the residuals of the models (normality and homogeneity of variances) were checked graphically. The existence of possible problems of multicollinearity between independent variables was verified with the variance inflation factor (VIF).

## RESULTS

Table 1 shows the characterization of the sample (53% boys) included in the study. There were no significant differences between the genders concerning age, body weight, BMI, or cardiorespiratory fitness (mL.kg^-^1.min^-1^). On average, boys had higher height values than girls.

**Table 1:**
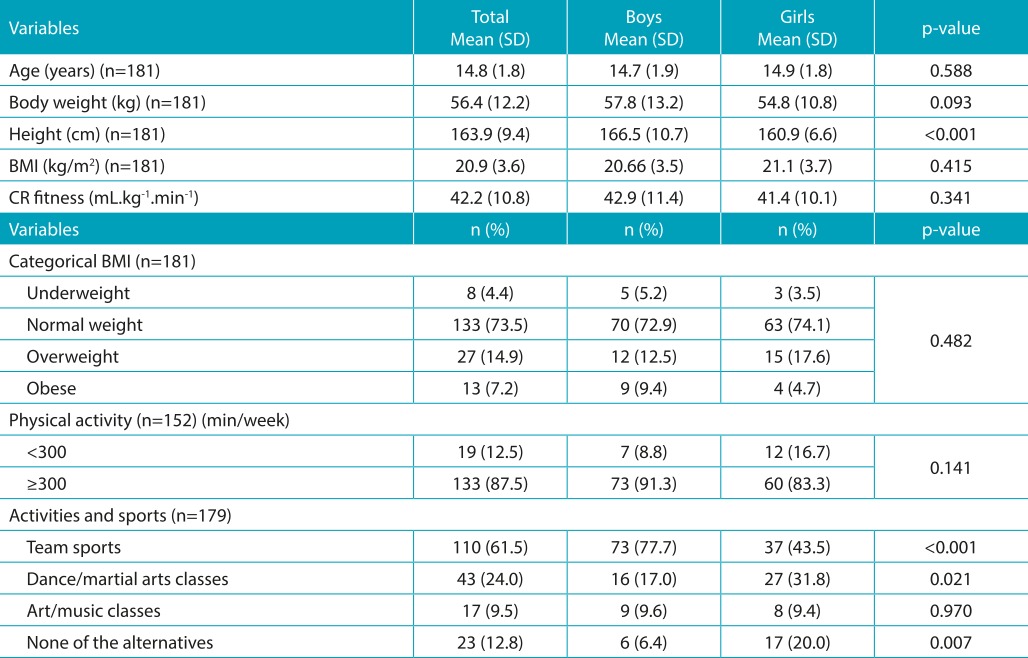
Description of the anthropometric, metabolic, and physical activity variables in adolescents from Ilhabela, São Paulo.

SD: standard deviation; BMI: body mass index; CR fitness: cardiorespiratory fitness.

Significant differences between the sexes have not been identified concerning transport to school, time spent sitting, or PA; 87.5% of adolescents achieved the PA recommendations. Significant differences between the sexes practicing team sports and participating in dance/martial arts classes were observed. The percentage of girls who were not engaged in any PA (20.0%) was higher than among boys (6.4%) (Tables 1 and 2).

**Table 2:**
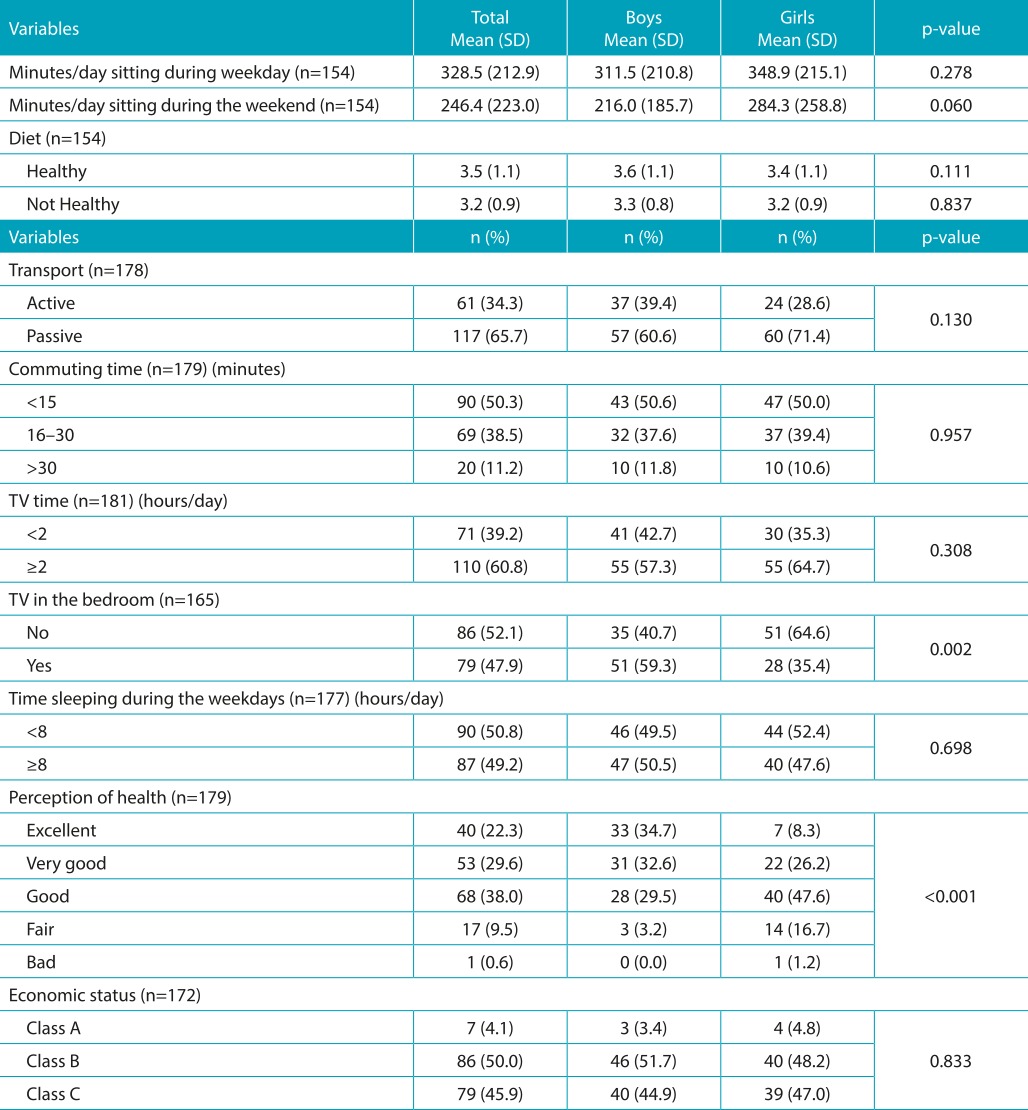
Description of the variables of lifestyle and economic status in adolescents from Ilhabela, São Paulo.

SD: standard deviation; TV: television.

The percentage of boys with TV in bedroom (59.3%) was significantly higher than among girls (35.4%). Approximately half of participants slept ≥8 hours/day (49.2%), and 45.6% of participants considered their sleep quality as very good. In regard to the amount of sleep, 44% rated it as good *(p>*0.05). Regarding the perception of health, most considered it as good (38.0%). Overall, the boys think they have better health in comparison with the girls (*p*<0.001) (Table 2).

There were no gender differences in the averages of healthy and unhealthy dietary habits indicators. Similarly, no differences were found between them in ES (Table 2).

The results of linear regression models to study the factors that influence cardiorespiratory fitness are shown in Table 3. The independent variables were those shown in Tables 1 and 2. Body weight was not considered due to the high correlation with BMI (*r*=0.854; *p*<0.001) and the creation of multicollinearity problems, when both variables are considered in the models (VIF>5).

**Table 3:**
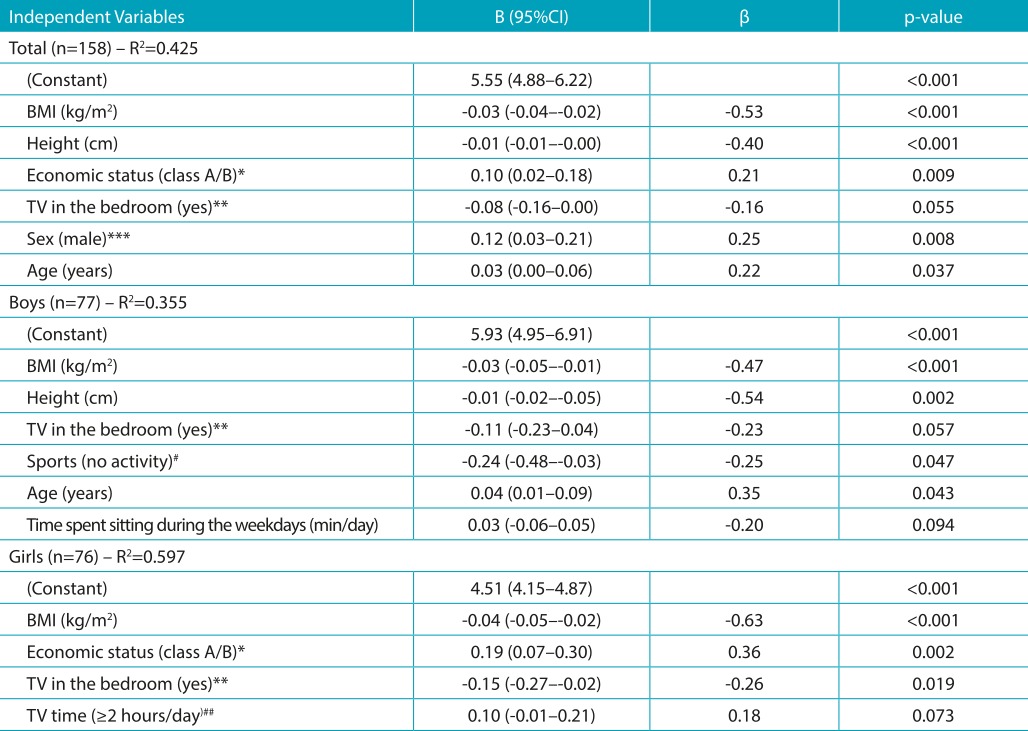
Linear regression models to verify the influence of lifestyle indicators on adolescent cardiorespiratory fitness according to sex.

R^2^: percentage of variability of the dependent variable, explained by the independent variables; Stepwise method: input criterion p<0.05; output criterion p>0.05; *effect in relation to class A; **effect in relation to those who do not have TV in the bedroom; ***effect in relation to girls; ^#^effect with respect to those who engage in some activity (collective sports, dance/martial arts classes, art/music classes); ^##^effect with respect to those who have TV time <2 hours/day; 95%CI: 95% confidence interval; BMI: body mass index; B: unstandardized regression coefficient; β: standardized coefficient

Table 3 shows that when the two sexes were analyzed together the variables with significant effects were BMI, height, ES, sex, and age. These variables explain in 42.5% (R^2^= 0.425) the variability in cardiorespiratory fitness. In boys, the variables with significant effect on cardiorespiratory fitness were BMI, height, lack of engagement with any sports activity, and age. These variables indicate in 35.5% (R^2^=0.355) the variability in cardiorespiratory fitness. In girls, the variables with significant effects were BMI, ES, and having a TV in the bedroom, explaining 59.7% (R^2^=0.597) of the variability in cardiorespiratory fitness (Table 3).

## DISCUSSION

The purpose of this study was to investigate the association of lifestyle indicators with cardiorespiratory fitness in adolescents from Ilhabela. In both sexes, lifestyle indicators (BMI, height, ES, sex, and age) had a significant effect on cardiorespiratory fitness. There was also a significant association of BMI, height, lack of engagement with any sports activity, and age with cardiorespiratory fitness among boys; and BMI, ES, and having TV in the bedroom among girls. This result corroborates other previous studies with adolescents in Brazil^23^ and other countries such as Portugal,^7^ which also found an association of lifestyle indicators (physical education classes, TV time,^23^ PA^7^) with cardiorespiratory fitness.

Research conducted in 12 countries showed that, in Brazil, 29% of boys and 15% of girls are obese (BMI Z score defined as >+2).^24^ This study revealed worrying data: 22% of adolescents were classified as overweight/obese, of which 9.4% of boys and 4.7% of girls were obese. This data is similar to that of the Nobre study,^25^ in which 24% of adolescents in the city of São Paulo were overweight/obese.

BMI proved to be a strong predictor of cardiorespiratory fitness both in boys (B=-0.03) and in girls (B=-0.04). These findings confirm those of a research study by Mello et al.^26^ Those authors^26^ demonstrated a significant association of low cardiorespiratory fitness (assessed by running ­test/­nine-minute walk) with high BMI value, regardless of gender. That data reinforces the importance of controlling body weight in adolescents; above the recommended weight is a detrimental factor in physical fitness.

Similarly to that reported by other authors,^26^ our results showed that when both sexes or only the female sex were analyzed, belonging to economic classes A/B positively influenced cardiorespiratory fitness when compared to those adolescents in class C. Vasques *et al* ^27^ found that adolescents of lower ES had lower levels of cardiorespiratory fitness when compared to those adolescents of higher ES. Having greater access to sports because of better financial conditions could explain this effect.

For Sisson *et al*,^28^ having a TV in the bedroom negatively impacts adolescent health. The equipment in the bedroom is positively associated with increased fat mass, increased waist circumference, subcutaneous fat, and being overweight. This study also found that girls who have a TV in the bedroom have lower levels of cardiorespiratory fitness. Thus, the TV in the bedroom seems to harm both body composition and cardiorespiratory fitness.

Sports are an important component for more daily energy expenditure and to promote a healthy lifestyle among adolescents.^29^ The study’s findings indicate that not being engaged in any team sport or extracurricular activity had both a negative and significant impact on the cardiorespiratory fitness of boys. Taliaferro *et al* ^30^ noted that participation in organized sports brings benefits to adolescent’s health, such as weight loss and a lower frequency of risky behavior. The relevance of these results in promoting PA and children’s health is justified by several factors, such as the recognized effect of PA on cardiovascular risk among children.^2^ ^,^ ^3^ ^,^ ^4^

For all lifestyle indicators analyzed in this study, the values of coefficients B found by linear regression models may not be considered high since cardiorespiratory fitness is determined by several factors, including body fat, sex, health status, age, genetics, and lifestyle.^7^ However, the data is worrying given the exposure of adolescents to cardiovascular risk factors during adolescence and adulthood. Future studies should investigate additional contextual factors that may help explain these associations and differences between the sexes. The associations of lifestyle indicators with cardiorespiratory fitness were different between the sexes, requiring further research in order to better clarify these associations and contribute to the discussion concerning the influence of lifestyle characteristics on biological variables.

These results suggest that the lifestyle impact on cardiorespiratory fitness in adolescents from Ilhabela may be related to sedentary behavior. Changes in lifestyle and maintaining regular PA influenced by parent initiatives and social support interventions are important strategies to address childhood obesity, physical inactivity, and large amounts of time being sedentary. However, other prospective studies are needed to determine the cause and effect relationship between lifestyle indicators and cardiorespiratory fitness, as some authors found an inverse relationship between sedentary lifestyle indicators and cardiorespiratory fitness.^5^ ^,^ ^6^ ^,^ ^7^ Therefore, the identification of risk groups is essential to the development of intervention strategies.

This study extends the existing literature on the association of several lifestyle indicators with cardiorespiratory fitness in adolescents. Nevertheless, the authors believe that the study has some limitations:

1. Cross-sectional design prevents the determination of the associations between cause and effect.
2. Non-representation of the sample limits the extrapolation of data for Brazilian schoolchildren.
3. The use of questionnaires to obtain lifestyle indicators.

The evaluation test for cardiorespiratory fitness, although submaximal and indirect, is suitable in nonhospital environments due to the reduced possibility of cardiorespiratory complications. The mean values of objectivity and reproducibility of cardiorespiratory fitness data ranged from moderate to high.^5^

It could be concluded that BMI, height, ES, sex, and age of the adolescents of both sexes influenced cardiorespiratory fitness. Among boys, the association was negative between BMI, height, and lack of engagement in any sports and cardiorespiratory fitness. On the other hand, age was positively associated. Among girls, cardiorespiratory fitness was negatively associated with BMI and TV in the bedroom, and positively associated with ES. Only BMI was common between the sexes. Intervention strategies and changes in lifestyle should be addressed differently between the sexes.
